# Supplementary material for: Structural and Functional Brain Abnormalities in Mouse Models of Lafora Disease
Source: Int J Mol Sci. 2020 Oct 20;21(20):7771. doi: 10.3390/ijms21207771 (PMC7589150; doi:10.3390/ijms21207771)
Supplement: Supplementary file 1 [file ijms-21-07771-s001.pdf]

Structural and functional brain abnormalities in mouse models of Lafora disease

Daniel F. Burgos[1#], Lorena Cussó[2,3,4,5#], Gentzane Sánchez-Elexpuru[1,6#], Daniel Calle[3,4], Max Bautista Perpinyà[1], Manuel Desco[2,3,4,5], José M. Serratosa[1], Marina P. Sánchez[1*]

Supplementary Figures

Supplementary Figure S1: VBM analysis of MRI in hippocampus and cerebellum of Lafora disease mice.

Supplementary Figure S2: Brain metabolite alterations observed *ex-vivo* by ^{1}H-HRMAS analysis in young *Epm2a*$^{-/-}$ and *Epm2b*$^{-/-}$ mice.

Supplementary Figure Legends

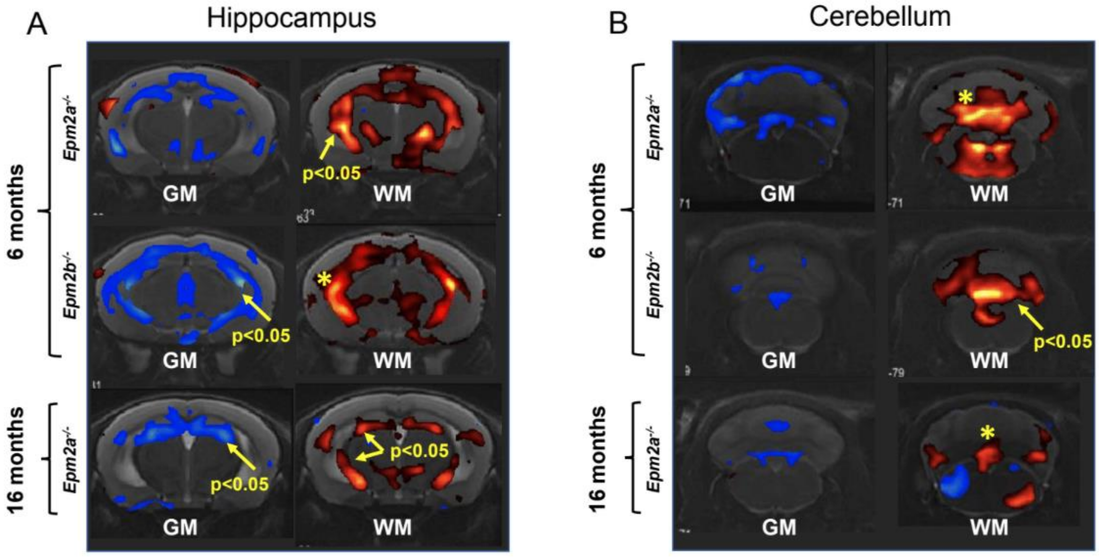

Figure S1. VBM analysis of MRI in hippocampus and cerebellum of Lafora disease mice. VBM of CSF, GM and WM indicating volume changes in images of hippocampus (A) and cerebellum (B) (amplified from Figure 2) in Lafora disease mouse models. Hot colors indicate increased and cold colors decreased regional volumes in *Epm2a*$^{-/-}$ and *Epm2b*$^{-/-}$ mice compared to control mice. In the hippocampus (A), a statistically significant decrease of GM volume was observed after FEW corrections in young *Epm2b*$^{-/-}$ and elder *Epm2a*$^{-/-}$ mice. Conversely, WM volume increase was shown in the hippocampus of all groups, with a statistically significant increase after FWE corrections in young and old *Epm2a*$^{-/-}$ mice. In the cerebellum (B), GM volume was not altered while WM volume increase was observed in all groups, with a statistically significance after FWE correction in young *Epm2b*$^{-/-}$ mice.

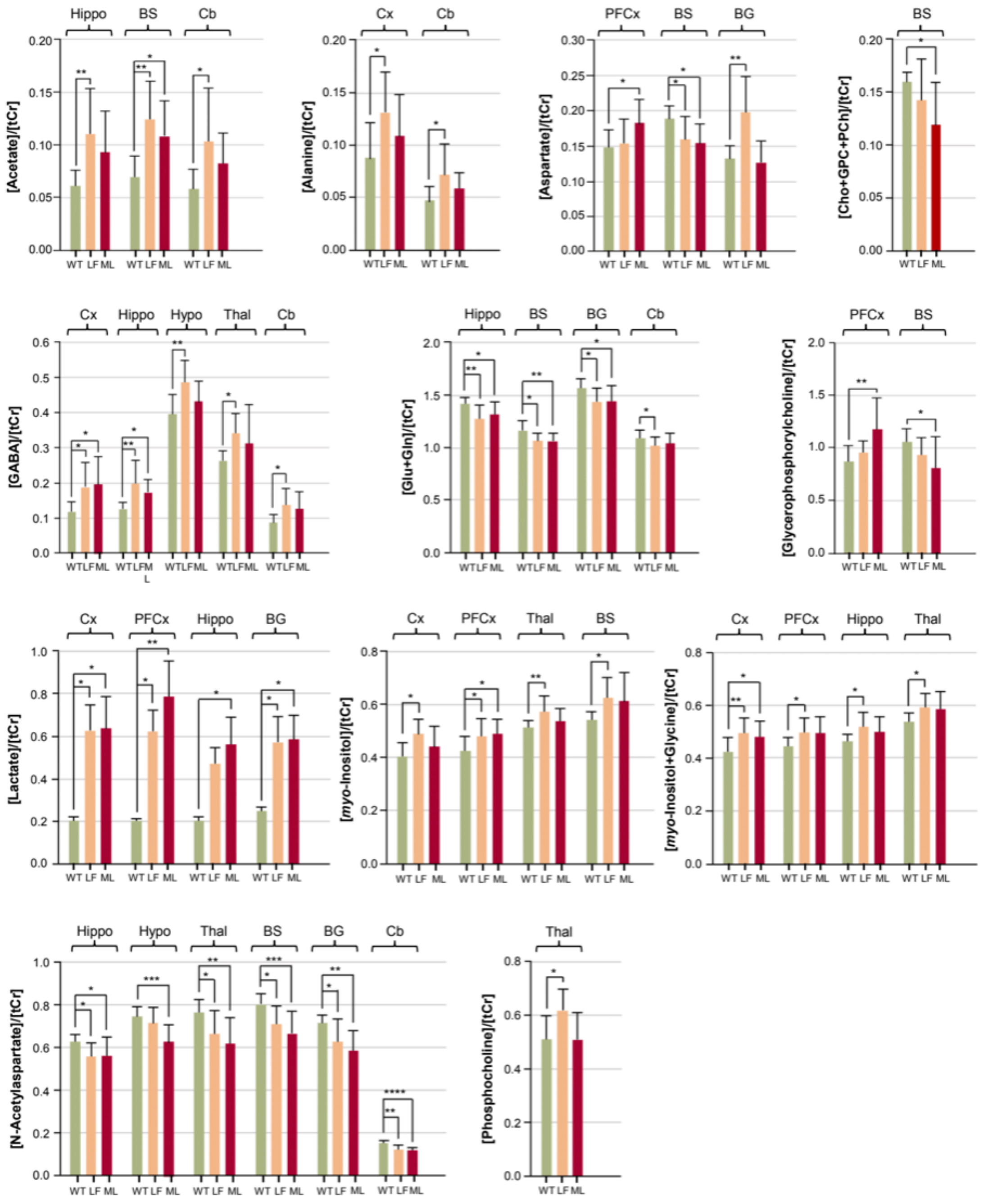

Figure S2. Brain metabolite alterations observed *ex-vivo* by ^{1}H-HRMAS analysis in 6-month-old *Epm2a*$^{-/-}$ and *Epm2b*$^{-/-}$ mice. Graphic representation of data collected in Table 1. Metabolites assessed were acetate (Ace), alanine (Ala), aspartate (Asp), choline (Cho), creatine (Cr), gamma-aminobutyric acid (GABA), glucose (Glc), glutamine (Gln), glutamate (Glu), glycine (Gly), glutathione (GSH), glycerylphosphorylcholine (GPC), lactate (Lac), leucine (Leu), *myo*-Inositol (*m*Ins), N-acetylaspartate (NAA), phosphatidylethanolamine (PE), phosphocholine (PCho), phosphocreatine (PCr), taurine (Tau), threonine (Thr), Cr from methylene protons (CrCH2), Cho+GPC+PCho, Cr+PCr, Glu+Gln, Lip13a, MM09, MM20, MM12, MM14, Lip20, Lip13a+Lip13, MM14,Lip13, MM09+Lip09, MM20+Lip20. Of all of them, only Ace, Ala, Asp, Cr, GABA, GPC, Lac, *m*Ins, NAA, PCho, PCr, Glu+Gln, *m*Ins+Gly and Cho+GPC+PCho showed significant altered values. In *Epm2a*$^{-/-}$ mice, Ace concentration normalized to tCr was statistically significant augmented in hippocampus, brainstem and cerebellum, while in *Epm2b*$^{-/-}$ mice, an increase of this

metabolite was only observed in brainstem (Fig. S2A). In *Epm2a*$^{-/}$ mice, an increase of Ala/tCr ratio was observed in cortex and cerebellum (Fig. S2B), with an increase of Asp/tCr ratio in basal ganglia and a decrease in brainstem (Fig. S2C). In *Epm2b*$^{-/-}$ mice, Asp/tCr increased in prefrontal cortex, while it decreased in brainstem (Table 1 and Supplementary Fig. S2C). GABA/tCr ratio augmented in cortex, hippocampus and hypothalamus of both models and also in thalamus and cerebellum of *Epm2a*$^{-/-}$ mice (Table 1 and Supplementary Fig. S2E). GPC/tCr ratio increased in prefrontal cortex and decreased in brainstem of *Epm2b*$^{-/-}$ mice, and it was not altered in *Epm2a*$^{-/-}$ mice (Table 1 and Supplementary Fig. S2F). Lac normalized to tCr increased in cortex, prefrontal cortex and basal ganglia of both models, and also in hippocampus of *Epm2b*$^{-/-}$ mice (Table 1 and Supplementary Fig. S2G). *m*Ins/tCr and *m*Ins+Gly/tCr ratios increased in cortex, prefrontal cortex and thalamus of *Epm2a*$^{-/-}$ mice, whilst *m*Ins/tCr also increased in brainstem of *Epm2a*$^{-/-}$ mice and in prefrontal cortex of *Epm2b*$^{-/-}$ mice. *m*I+Gly/tCr ratio increased in hippocampus of *Epm2a*$^{-/-}$ mice and in cortex of *Epm2b*$^{-/-}$ mice (Table 1 and Supplementary Fig. S2H and N). NAA/tCr decreased in all regions analyzed of *Epm2a*$^{-/-}$ and *Epm2b*$^{-/-}$ mice, with the exception of cortex and prefrontal cortex (Table 1 and Supplementary Fig. S2I). PCho/tCr decreased in thalamus of *Epm2a*$^{-/-}$ mice (Table 1 and Supplementary Fig. S2J) and Cho+GPC+PCh/tCr decreased in basal ganglia of *Epm2b*$^{-/-}$ mice (Table 1 and Supplementary Fig. S2L). Glu+Gln/tCr ratio decreased in hippocampus, brainstem and basal ganglia of both models and in cerebellum of *Epm2a*$^{-/-}$ mice (Table 1 and Supplementary Fig. S2M). Cortex (Cx), prefrontal cortex (PFCx), hippocampus (Hippo), hypothalamus (Hyp), thalamus (Thal), brainstem (BS), basal ganglia (BG) and cerebellum (Cb). *$p < 0.05$; **$p<0.01$; ***$p<0.001$.
